# Compositional cyber-physical epidemiology of COVID-19

**DOI:** 10.1038/s41598-020-76507-2

**Published:** 2020-11-11

**Authors:** Jin Woo Ro, Nathan Allen, Weiwei Ai, Debi Prasad, Partha S. Roop

**Affiliations:** 1 Department of Electrical, Computer and Software Engineering, University of Auckland, Auckland, New Zealand; 2 Faculty of Medical and Health Sciences, University of Auckland, Auckland, New Zealand

**Keywords:** Epidemiology, Biomedical engineering, Electrical and electronic engineering

## Abstract

The COVID-19 pandemic has posed significant challenges globally. Countries have adopted different strategies with varying degrees of success. Epidemiologists are studying the impact of government actions using scenario analysis. However, the interactions between the government policy and the disease dynamics are not formally captured. We, for the first time, formally study the interaction between the disease dynamics, which is modelled as a physical process, and the government policy, which is modelled as the adjoining controller. Our approach enables compositionality, where either the plant or the controller could be replaced by an alternative model. Our work is inspired by the engineering approach for the design of Cyber-Physical Systems. Consequently, we term the new framework Compositional Cyber-Physical Epidemiology. We created different classes of controllers and applied these to control the disease in New Zealand and Italy. Our controllers closely follow government decisions based on their published data. We not only reproduce the pandemic progression faithfully in New Zealand and Italy but also show the tradeoffs produced by differing control actions.

## Introduction

The ongoing Coronavirus Disease 2019 (COVID-19) presents an unprecedented global crisis with 30,675,675 infections and 954,417 deaths as of 20th September 2020^1^. There are now widespread calls for new techniques for intervention, including methods of rapid testing even at the home^2^. While Epidemiologists are studying the dynamics of the diseases using computational models, governments are trying to “flatten the curve”^3^ to reduce the health impacts. This is achieved through Nonpharmaceutical Interventions (NPIs), such as lockdowns and social distancing methods ^4^.

The Majority of the research focus in epidemiology has been on mathematical modelling of the disease dynamics. Many governments, like the New Zealand government, have also worked closely with the scientific community^5^ to arrive at critical decisions. But how can we ascertain which model is better, and in which settings^6^? There exists no clear methodology to formally *capture and classify criteria-based actions of the government*^7^ as mathematical models. Also, *given the wide variability of government actions globally, how can we formally assess them while studying their impact?* This paper tries to provide answers to these questions for the first time, by focusing on the compositional modelling of government actions alongside an epidemiological model of the disease.

While at the policy level there has been minimal engineering thinking to provide solutions, it is evident that the pandemic and its control bear many similarities with the well known engineering domain of Cyber-Physical Systems (CPSs)^8,9^. In a CPS, a physical process such as the electrical conduction of the human heart (known as the *Plant*) is controlled by an adjoining device such as a pacemaker, also known as a *Controller*^10^. This closed-loop system mimics the behaviour of a piece-wise continuous phenomena, where the plant’s dynamics is modelled using a set of Ordinary Differential Equations (ODEs). The plant makes discrete mode switches based on the actions of a discrete controller.

**Figure 1:**
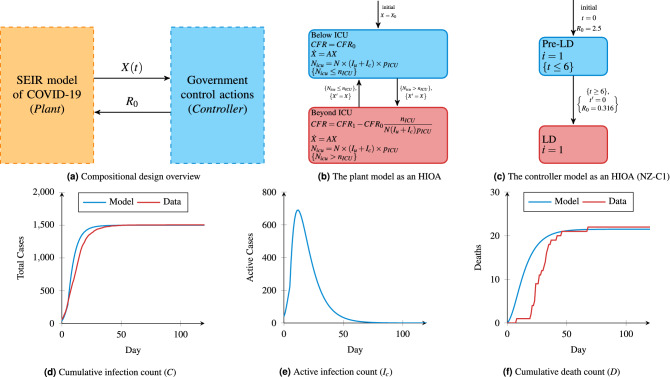
The proposed compositional design of Compositional Cyber-Physical Epidemiology and simulation results. For (**d**–**f**), day 0 corresponds to 20th March 2020. For (**d**,**f**) these numbers are compared with the available New Zealand data.

In the setting of COVID-19, we may view the plant as the dynamics of disease progression, already modelled faithfully using several epidemiological models^11,12^. The adjoining controller is a state machine that can induce mode switches in the plant. Such a closed-loop system may be depicted as shown in Fig. 1a and we term this approach Compositional Cyber-Physical Epidemiology (CCPE). Here, the plant provides the state of the pandemic encapsulated as a vector of variables *X*(*t*), while the controller affects the state of the plant by trying to alter the value of the reproduction number $R_0$, which represents the average number of new infections for each infectious person.

There is recent evidence that such engineering thinking may have relevance for COVID-19. The Institute of Electrical and Electronics Engineers (IEEE) published an article citing the benefits of the application of such feedback control theory^13^, which is evidence of concurrent thinking along our lines. However, their work is primarily based on studying the impact of fictitious controllers over a simple disease model, without considering the actual data from the current pandemic.

This paper advocates that a compositional design approach is needed to include the NPI techniques with the existing epidemiological models. Such an approach, which we term *CCPE*, would allow for the creation of more realistic models which can answer more questions than any existing individual model^6^. This can be used in the decision making of a government, with the goal of both minimising the death toll while reducing the economic impact of any restrictions.

We use Susceptible, Exposed, Infected, Removed (SEIR) model^11,12^ for the illustration of our methodology, while stressing that the developed methodology is amenable to any other dynamical model based on ODEs. The SEIR model incorporates coupled ODEs, and has been utilised previously in the context of COVID-19^14^. These ODEs capture the progression of a disease through the population, as people become infected, progress through their infection, and infect others. SEIR models include variables which represent the population during an epidemic which can be in a range of states: susceptible (*S*), exposed (*E*), pre-symptomatic (*P*), infectious (*I*), recovered (*R*) and deaths (*D*). The infected and recovered cases are further categorized into untested ($I_u$, $R_u$) and confirmed cases ($I_c$, $R_c$) to enable control mechanisms which are specific to confirmed cases. The key parameter determining if a virus can cause an epidemic is the reproduction number $R_0$ and depends on both the transmissibility of the virus and social distancing. For $R_0>1$ the virus will spread until herd immunity has been established, while for $R_0<1$ the transmission will progressively decay until the virus is eradicated^12^. In addition to $R_0$, further parameters are used for capturing aspects such as the fatality rate and testing rate.

Government interventions can be used to modify each of these parameters such as the use of NPIs to reduce the reproduction number, or increased testing to isolate more confirmed cases. These responses vary between countries and typically vary over time depending on the local situation^2,15^. For example, New Zealand acted with a “hard and fast” response that quickly implemented quarantines and immigration restrictions, similar to other countries like Vietnam^16^. The New Zealand response implemented an alert system for COVID-19^17^ which comprises four levels of increasingly strict interventions. In this case, the four levels can be modelled as a discrete controller^9^ which can interact with the continuous SEIR model as a type of CPS. Such an approach can be used in order to continuously evaluate the efficacy of the control strategy while the pandemic is still in a relatively nascent period and more information is being learned about its dynamics each day.

The use of formal modelling for biological processes has been advocated by Fisher and Henzinger^18^, which makes a distinction between computational models and *executable models*. More recently, Bioengineers have adopted an executable model called Hybrid Input-Output Automata (HIOAs)^10,19^ for developing abstract models. These abstractions are used to achieve behaviour from cellular^20^ to organ levels^21,22^. These abstract models are also “executable” in the sense that hardware and software implementations may be derived from them so that they work as virtual organs^21,23,24^.

An HIOA captures both the continuous (i.e. the population model) and discrete (i.e. the government controller) dynamics through the use of an automata with included ODEs. The conversion of the SEIR model into HIOA results in the formal model of Fig. 1b, where the two locations capture whether the Intensive Care Unit (ICU) capacity has been exceeded. The formal nature of these models means that they can be used in simulation and code generation frameworks with relative ease^25,26^. We have used the recently developed Intensive Care Unit (ICU)^23^, which allows for the specification of a complex network of HIOAs.

To illustrate our methodology we have selected New Zealand and Italy, who have adopted disease management approaches that differ in terms of their response speed—where New Zealand is quick to increase restrictions, Italy takes comparatively longer. We show that in the case of the four-level New Zealand control, we are able to make decisions around the optimal criteria for switching between the control modes to minimise the impact of the virus. Our methodology is generic enough and has the potential to be adopted to other alternative settings.

## Results

### A simple controller in the New Zealand context

The proposed CCPE approach is first demonstrated using the New Zealand COVID-19 context using a simple controller we term NZ-C1. The NZ-C1 control strategy is to initiate a strong lockdown measure, which is introduced early and is not lifted until the new infections approach zero. We compare against the official data which contains the number of cases (both confirmed and probable), recovered, and deaths for every day from 20th March 2020 to 9th September 2020. Also, on 26th March 2020 (6 days after the first date in our data), the New Zealand government initiated their full lockdown measures, which remained in place until 28th April 2020 when some restrictions began to be eased.

First, we examine the accuracy of our CCPE approach by comparing it to the New Zealand data as a means to increase confidence in our predicted future disease dynamics, as per Fig. 1. In our framework, we propose the modelling of both the plant and controller as HIOA^9^. We create a simple controller (Fig. 1c), which transitions into a lockdown mode (LD) 6 days after the start. The controller modes are depicted as two different states of the system, namely Pre-LD and LD respectively. Within every mode, we encapsulate a condition that determines the maximum time control can reside in a given mode, which is known as the *invariant*. In the LD mode, however, no such invariant is specified. In this case, the invariant is by default true and hence control can remain in this location forever. In contrast, control can remain in the Pre-LD mode, when the current time *t* is less than 6 days. The rate of change of time is modelled as ODE ${\dot{t}}=1$ within both modes.

Transitions between modes happen when some conditions are satisfied. For example, the transition from Pre-LD to LD happens when the current value of *t* becomes 6. When a transition triggers, some variables are updated. For example, when this transition triggers, the value of time *t* is reset (by the reset action, which is denoted $t'=0$). Also the value of $R_0$ is set to 0.316.

For this model, we use the previously described values for $R_0$ of 2.5 and 0.316 for pre-lockdown and lockdown respectively. Figure 1d–f show the results of this simulation for three main metrics. By day 120 (19th July 2020) the cases have levelled out in both the real data and model data. On this date, the simulated model predicts 1505 confirmed cases, while in reality there were 1506, an error of only 1 case. Overall, the correlation coefficient is 0.9752.

With this simple controller which remains in lockdown indefinitely (i.e. until a vaccine arrives), the cumulative infection count converges to a realistic number, indicating that the 4 weeks of lockdown was effective at eraditcating the disease. This can be seen in Fig. 1e, where the active infections decrease to around zero by day 100, meaning that the disease has been eradicated. Finally, the total number of deaths in this scenario is expected to be 22, which matches up exactly with the official data.

### CCPE model of the New Zealand Government control strategy

**Figure 2:**
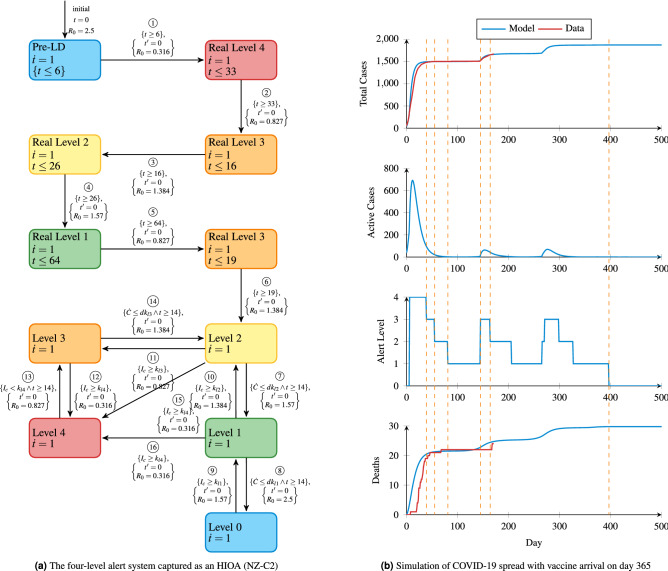
The controller and simulation results corresponding to the New Zealand system for fighting COVID-19.

Next, we investigate the disease dynamics in New Zealand over a longer period of time (600 days) with a more complex model which closely follows the government’s strategy of four different levels of increasing stringency, along with their historical changes. The previous controller (Fig. 1c) is extended by incorporating a control policy that reflects these alert levels, where Pre-LD and LD correspond to Level 0 and Level 4 respectively. This new controller is called NZ-C2 and is shown in Fig. 2a. Additionally, the plant model is augmented to allow for the introduction of a “second wave” of infections around day 100 to capture what was experienced in the New Zealand context.

Until 9th September 2020 (day 172) NZ-C2 follows the historical actions of the real-world control strategy. Subsequently, NZ-C2 tries to set the alert level in order to determine an appropriate reproduction number $R_0$ for the current situation. We have based our work on the reports released by the New Zealand government and the analysis of $R_0$ values and associated alert levels^7^.

For New Zealand, Table 2 presented in the “Methods” section lists major interventions and their associated relative reproduction number changes indicating how they increase/decrease the $R_0$. According to^27^, the initial value of $R_0$ is 2.5 without any control, which corresponds to alert level 0 in our model. In summary, the $R_0$ values for alert levels 4 through 1 are 0.316, 0.827, 1.384, 1.570, respectively. The maximum value of $R_0$ is 2.5, which corresponds to level 0.

The controller HIOA which captures the transitions between these levels is shown in Fig. 2a. Here, the conditions for increasing the alert level are based on the current number of infected cases ($I_c$). For example, from level two if $I_c \ge k_{l3}$ then the alert level immediately rises to three. On the other hand, the alert level can go down if the increasing rate of new cases per day (${\dot{C}}$) is less than a certain amount. For example, from level three if ${\dot{C}} \le dk_{l3}$ then the alert level decreases to level two. In addition, to avoid frequent oscillations between levels, a minimum duration within a level before being able to drop down to a lower level is added and is set to be 14 days.

The simulation results for this controller are shown in Fig. 2b, where we also include the presence of a vaccine from day 365, which results in the country moving out of all remaining lockdown measures on day 397 (22nd April 2021). In contrast to the scenario of continuing the lockdown based on the previous controller NZ-C1, we observe gradual step downs in the control level. Although there will be 30 deaths, 8 more than the previous lockdown scenario, the four-level approach allows for society to begin its return to normalcy from day 39 in order to minimise economic damage relative to the controller NZ-C1 caused as a result of enforced social distancing^4^.

Additionally, the model and controller show their ability to capture both the real-world second wave (at around day 150—18th August 2020)) in a realistic manner, as well potential future outbreaks (from around day 250—18th August 2020)). In these cases, the controller reacts proportionately to the risk associated with the current situation based on both the number of active cases ($I_c$) and the rate of new cases (${\dot{C}}$).

### Modelling Italy’s control strategy

**Figure 3:**
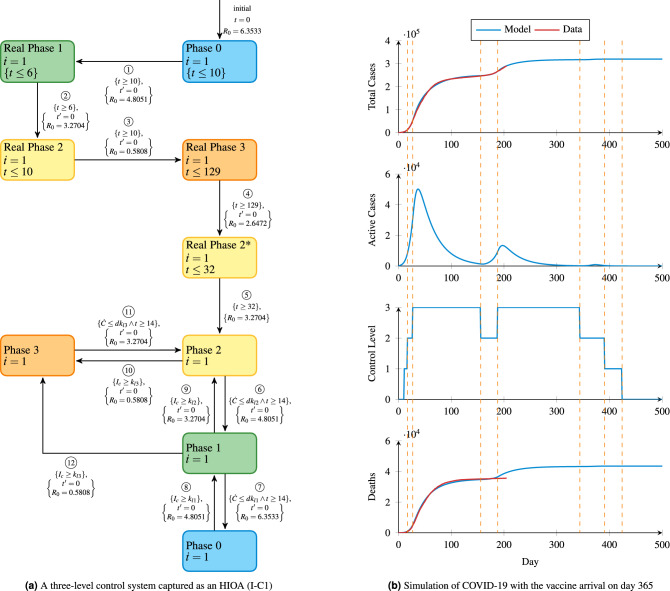
The controller and simulation results corresponding to the Italy system for fighting COVID-19.

The CCPE approach can be adapted to the intervention techniques of other countries. For example in Italy, the government does not have an explicitly outlined intervention system, instead the control actions are progressively released as they are needed. We use the published *stringency index*^15^ for Italy across time in order to create an approximation of their control strategy in our framework. For example, on 23rd February 2020, the stringency index was listed as 66.67, while subsequent measures increased this to 71.43, 90.48, and finally 95.24^15^. We create an approximate discrete controller for this approach (Fig. 3a), where the phases correspond to a degrees of stringency mentioned earlier. Note that the date of first observation point in the Italy data is 23rd February 2020.

The control flow of the Italy model called I-C1 in Fig. 3a is as follow. From the initial state Phase 0, transition $\textcircled{1}$ leads to Phase 1. This transition is triggered based on time, according to the historical actions of Italy government. For instance, Italy was in Phase 0 on 23rd February 2020, and moved to Phase 1 by closing the schools and universities on 4th March 2020. Similarly, transitions $\textcircled{2}$, $\textcircled{3}$, $\textcircled{4}$, and $\textcircled{5}$ are triggered based on the time when historical actions were imposed. A countrywide lockdown was issued on 10th March 2020 (day 16) and the nation entered Phase 2. On 20th March 2020 (day 26), the government further tightened the control by reducing the public transportation and initiated Phase 3, before beginning to relax restrictions on 27th July 2020 (day 155) which allowed for a second wave of infections. For Italy, according to our estimation based on^15^, the reproduction number $R_0$ is 6.3533 in Phase 0, 4.8051 in Phase 1, 3.2704 in Phase 2 (2.6472 in Phase 2*), and 0.5808 in Phase 3.

After the historical transitions have been performed ($\textcircled {1}$—$\textcircled {5}$) in Fig. 3a, we apply the same control strategy presented in Fig. 2a. That is, the control level can decrease based on $\dot{C}$ and time remained in a level, or the control level can increase based on $I_c$. Precisely, the same level changing conditions are used for Italy. In this way, we can examine the performance of the same controller in different countries. We set 10, 5, and 0.01 for $dk_{l3}$, $dk_{l2}$, and $dk_{l1}$, respectively. Also, $k_{l3}$, $k_{l2}$, and $k_{l1}$ are 6046, 3023, and 605, respectively. Additionally, the constraint to level four ($k_u$) is equal to the hospital capacity of approximately 483,694^14^.

The simulation results for the Italy model are shown in Fig. 3b. When control goes down to phase two for the first time, the active infection count ($I_c$) starts to increase again, causing a second wave of infections and necessitating the return to phase three. We can observe that in order to deal with this second wave, the controller implements phase three and remains there until day 344. Overall, the simulation predicts that approximately 45,000 deaths and 320,000 confirmed cases are expected in Italy.

**Figure 4:**
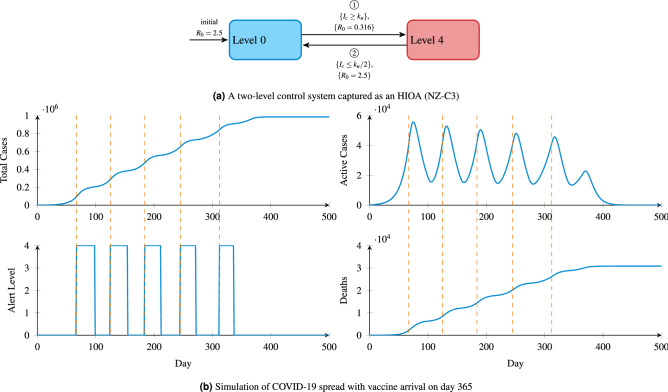
Examples of government interventions with only lockdown action.

### Modelling other controllers

We can examine various “what if” scenarios of COVID-19 in New Zealand, as a result of varying intervention techniques. A simple control policy in previous work has consisted of only two levels, essentially a full lockdown and no control^14^. Precisely, a complete lockdown (level four) is triggered if the currently active infection count exceeds the hospital ICU capacity ($\{I_c \ge k_u\}$), while in times where the currently active infection count is less than the half of the hospital capacity ($\{I_c \le k_u/2\}$), the lockdown is removed (level zero), as shown in Fig. 4a.

The simulation results for this model in our framework are shown in Fig. 4b. As expected, the control level frequently switches between four and zero as the number of active cases oscillates. This results in a number of 1-month lockdown happening after day 67. Although the peaks of oscillation in the infection case graph gradually diminishes over time, the final number of deaths is extremely high and control remains in the lockdown for a long period of time, causing drastic impacts on the economy.

## Discussion

The compositional approach of CCPE allows the flexibility of formal modelling and validation of government control strategies to manage a pandemic. We have shown the ability of CCPE to model the dynamics of COVID-19 in conjunction with the various intervention techniques that governments employ. Table 1 compares the controllers used in this paper. As we can see, in the case of New Zealand, the controller NZ-C2 achieves much better overall outcome compared to the simple controller NZ-C1. While the lockdown for NZ-C1 lasts until a vaccine is available, the economic impact of such a long lockdown may be catastrophic. In contract, the controller NZ-C2 has a gradual lifting of restrictions, which reaches level 1 much faster. Also, the overall risk of this strategy is a marginal increase in the number of deaths. In contrast to these two controllers, is the third control strategy NZ-C3, which introduces oscillations. We can see immediately the impact of a poorly managed control strategy, which may lead to three orders of magnitude more deaths. Finally, we also present the controller for Italy, which is modelled based on the actions of their government and as reported in^15^.

CCPE allows the formal modelling of complex controllers. This enables the systematic evaluation of various control strategies in order to determine the best approach for a country which minimises the economic and social impacts, in addition to achieving the best healthcare outcome. As long as each NPI can be quantified, in terms of $R_0$ or testing rate, then the importance of individual NPIs can also be evaluated in terms of their overall impact on pandemic control. These NPIs can then be organised into an overall policy which includes both the NPIs to enforce, the conditions under which they are enforced, and how long they should remain in place.

**Table 1:** A summary of Compositional Cyber-Physical Epidemiology case studies.

Figure	Plant	Controller	Confirmed cases	Deaths	Description	Social impact
1	PL-2	NZ-C1	1501	22	Indefinite lockdown	Lockdown lasts until vaccine is available
2	PL-3	NZ-C2	1874	30	Four level control	Business can operate after day 39, and a near zero infection count is achieved on day 397
4	PL-1	NZ-C3	983,960	30,835	Two level control	Infection count oscillates until it reaches zero on day 481
3	PL-1	I-C1	319,834	43,540	Three level control	A near zero infection count is achieved after 424 days

While the CCPE framework as presented here is based on the SEIR model^14^, there is nothing that restricts our framework to such a model. Any continuous model which can be captured through a series of ODEs is able to be used which can open the door to more accurate simulation techniques, such as the enhanced version used by CovidSIM^5^, the recent SIDARTHE model^28^, or even microscale modelling. We have already shown this ability by suggesting some modification to SEIR to better account for contact tracing and isolation. This is presented in “Methods” section and is denoted as the revised plant model PL-2 in Table 1. Additionally, PL-3 allows for the capturing of the second wave dynamics.

The effectiveness of the CCPE framework relies on the fidelity of the transmission model and parameter estimation, requiring expertise in both epidemiology and statistical analysis, both of which are rapidly changing as our understanding of the pandemic increases. As such, the estimation of $R_0$ is technically challenging^29^ and the value varies due to different model assumptions and estimation procedures^27,30–33^. While the World Health Organization (WHO) estimates that the basic $R_0$ ranges between 1.4 and 2.5^34^, Liu et al. suggested that the value is expected to be higher based on evolving research^35^. To further complicate this matter, cultural differences between countries can have an impact on the effectiveness of NPIs at reducing $R_0$, such as social distancing^4^.

To apply the CCPE framework to other countries, the $R_0$ value should be examined. However, this reproduction number varies based on the control measures implemented by each country^29,30,36–38^. To investigate the interaction between government interventions and disease transmission dynamics, action-specific $R_0$ values are essential. Apart from the control actions, many factors, such as population density^39^, mobility^30^, and spatial heterogeneity^40^, affect the $R_0$ value.

The ability for our CCPE framework to work across a range of these different country-specific plant models and various control designs creates a useful tool for designing strategies to fight COVID-19. The analysis of counter-measures and their impact on dealing with the disease has traditionally been limited to simple “if-else” style controllers, and here we show the ability to model counter-measures which are able to include some form of state in their logic.

## Outlook

In this work, we evaluated the composition of a controller with an epidemiological model. However, the CCPE framework is far more flexible. HIOA-based modelling can be composed with any number of other HIOA. Further HIOAs could be used which take into account aspects such as legislation, culture, economy structure, administration, etc.^4,41–43^. For example, an economic model could be added^3^, which takes into account the various measures being applied in order to provide a metric of the financial toll. Such a model could then be used to design a controller which not just minimises the number of deaths in the population, but also reduces the economic impact in a form of bi-criteria optimisation^44,45^.

In our work, the criteria used for switching between modes of the controller were based on comparing the number of active cases to the ICU capacity. Instead, control mechanisms could be created which take into account additional information, such as the climate, to more accurately capture the decision-making process. Moreover, we could further refine the dynamical modes to better represent the rate of testing.

Finally, a robust estimation approach of action specific $R_0$ values within context of geographical and social heterogeneity should be systematically investigated in the future. COVID-19 is still relatively new and there exists a large variation in potential reproduction numbers between studies. We note that the accuracy of any epidemiological model depends on the accuracy of its reproduction number, and so further improvements in this area would be of great benefit. For example, there is the potential for the adoption of an approach as recently proposed in^13^ if the reproduction number could be approximated as a continuous function. While this is a challenging proposition, our work opens the door for more engineering researchers to create an impact on current and future pandemics. A momentum is already in evidence as reported in^46^ to show how Engineers are coming together to contribute to this cause in various ways.

## Methods

### The SEIR model of COVID-19

The modified SEIR model^14^ consists of variables which represent the various sub-populations during an epidemic: susceptible (*S*), exposed (*E*), pre-symptomatic (*P*), infectious (*I*), recovered (*R*), and deaths (*D*). The infectious and recovered cases are further categorized into untested ($I_u$, $R_u$) and confirmed ($I_c$, $R_c$). The dynamics of the transmission between these sub-populations can be described by a series of coupled ODEs, shown in Eq. (1) through (8).

1 $$\begin{aligned}&\frac{\mathrm {d} S}{\mathrm {d} t}=-\beta S(\epsilon P+I_u+I_c) \end{aligned}$$

2 $$\begin{aligned}&\frac{\mathrm {d} E}{\mathrm {d} t}=\beta S(\epsilon P+I_u+I_c)-\alpha E \end{aligned}$$

3 $$\begin{aligned}&\frac{\mathrm {d} P}{\mathrm {d} t}=\alpha E-\delta P \end{aligned}$$

4 $$\begin{aligned}&\frac{\mathrm {d} I_u}{\mathrm {d} t}=\delta P-(\gamma +c)I_u \end{aligned}$$

5 $$\begin{aligned}&\frac{\mathrm {d} I_c}{\mathrm {d} t}=cI_u-\gamma I_c \end{aligned}$$

6 $$\begin{aligned}&\frac{\mathrm {d} R_u}{\mathrm {d} t}=\gamma (1-CFR)I_u \end{aligned}$$

7 $$\begin{aligned}&\frac{\mathrm {d} R_c}{\mathrm {d} t}=\gamma (1-CFR)I_c \end{aligned}$$

8 $$\begin{aligned}&D=1-S-E-P-I_u-I_c-R_u-R_c \end{aligned}$$

The Case Fatality Ratio (CFR) depends on the number of active people in the ICU and the ICU capacity. If the number of active people in the ICU is within the ICU capacity then the CFR is is simply equal to some lower bound $CFR_0$ (1%). When this limit is exceeded, the CFR is decided by a mixture of patients who are receiving ICU care ($CFR_0$) and those who are not ($CFR_1$). The result of this is a piecewise function as in Eq. (10) where $CFR_1$ (2%) is the maximum fatality rate, $n_{ICU}$ denotes the maximum ICU beds, *N* is the population size, and $p_{ICU}$ is the proportion of total cases which require ICU attention.

9 $$\begin{aligned}&patients = N \times (I_u + I_c) \times p_{ICU} \end{aligned}$$

10 $$\begin{aligned}&CFR = \left\{ \begin{array}{cl} CFR_0 &{} \text {if}\ patients \le n_{ICU} \\ CFR_1 - (CFR_1 - CFR_0) \times \dfrac{n_{ICU}}{patients} &{} \text {otherwise} \end{array}\right. \end{aligned}$$

The SEIR model can be described as HIOA, shown earlier in Fig. 1b. *X* is the vector of all epidemic variables initialized to $X_0$, *A* is the matrix of the parameters, and ${\dot{X}}=AX$ is the matrix representation of Eqs. (1) through (8)). When the ICU demand $N_{icu}$ is less than or equal to the maximum ICU capacity $n_{ICU}$, the HIOA stays in the location *Below ICU* with CFR of $CFR_1$. Otherwise, control goes to the location *Beyond ICU* and the CFR is defined by Eq. (10).

In the model (Eq. 1, 2 ), the reproduction number $R_0$ determines the transmission rate $\beta$ as per Eq. (11). Here, $\epsilon$ is the relative infectiousness in the presymptomatic period, $\delta$ is the transition rate from presymptomatic to infectious, and $\gamma$ is the transition rate from infectious to recovered.

11 $$\begin{aligned} \beta =\dfrac{R_0}{\epsilon /\delta +1/\gamma } \end{aligned}$$

These transition rates are decided by the virus nature, while $R_0$ depends on the contacts and the transmissibility^12^. The government control measures can impact this reproduction number, and hence also $\beta$, by reducing:

- physical contacts (e.g. travel restriction, self-isolation, work at home, close schools, etc.), or
- the transmissibility (e.g. hand washing, public disinfection efforts, etc.)

In order to start the propagation of the disease through the population we start with an initial number of cases ($I_c$) which matches with the initial number of reported cases. Typically, our simulations start after a country has reached 100 total cases as this is a likely point where local transmission, if not community transmission, has started to occur. Additionally, this allows us to isolate the population from the rest of the world and ignore the potential inflow and outflow of infected people as travel is heavily restricted by this point in time.

We propose a revision of the recent SEIR model^14^ in this paper to account for better management of the pandemic using improved case isolation and contact tracing. In Table 1, we denote the SEIR model^14^ as the plant model PL-1 while our revised model is marked as the plant model PL-2. This is since case isolation and contact tracing could significantly reduce $R_0$ for identified cases (i.e. $I_c$)^37^. We use different parameters for the transmission rate $\beta$ (Eqs. 1, 2 ) such that the confirmed cases have lower transmissivity due to the combined effects of isolation and contact tracing. The resultant refined model replaces Eq. (1) and (2) with Eq. (12) and (13) .

12 $$\begin{aligned}&\frac{\mathrm {d} S}{\mathrm {d} t}=-\beta _1 S(\epsilon P+I_u)-\beta _2 S I_c \end{aligned}$$

13 $$\begin{aligned}&\frac{\mathrm {d} E}{\mathrm {d} t}=\beta _1 S(\epsilon P+I_u)+\beta _2 I_c-\alpha E \end{aligned}$$

Further, to capture the the dynamics of externally introduced second waves we create a further extended plant model named PL-3. This model, at some predefined time $t_2$ will instantly move a set number of people from the susceptible population (*S*) into the exposed population (*E*). Such a model is required in the context of New Zealand, where it is most likely that eradication of the virus was achieved prior to the emergence of the second wave and thus the cause of this re-emergence was most likely external.

### The New Zealand model of COVID-19

For the epidemiological model of New Zealand, we use base reproduction number of 2.5 as is widely reported without control measures in place^27^. To investigate the interaction between government interventions and disease transmission dynamics, we need to introduce various reproduction numbers for the different action control strategies and stages. The estimation of $R_0$ is technically challenging^29^ and a number of studies have been done^31,32,35,38,47^. However, these values are not specific to certain control policies.

**Table 2:** A list of the interventions involved at each alert level in New Zealand, and the reproduction number derivation. A tick (✔) represents that an intervention is applied, a cross (✗) means that it is not applied, and a triangle (△) is used when an intervention is partially applied.

Intervention	Weight	Level 4	Level 3	Level 2	Level 1	Level 0
Widespread testing	0.186	✔	✔	✔	✔	✗
Temperature checkpoints	0.093	✔	✔	✔	✔	✗
Contact tracing	0.186	✔	✔	✔	✔	✗
Close contacts of confirmed cases ordered to self-isolate	0.093	✔	✔	✔	✔	✗
Large scale disinfection efforts	0.046	✔	✔	✔	✗	✗
Distribution of PPE to at-risk workers	0.093	✔	✔	✔	✔	✗
Hygiene public awareness efforts	0.186	✔	✔	✔	✔	✗
International travel ban	0.186	✔	✔	$\bigtriangleup$	✗	✗
Domestic travel restrictions	0.093	✔	✔	$\bigtriangleup$	✗	✗
People forced to remain home	0.186	✔	✗	✗	✗	✗
Bans on outdoor gatherings over 500 people	0.093	✔	✔	✔	✔	✗
Bans on indoor gatherings over 100 people	0.093	✔	✔	✗	✗	✗
Bans on recreational sports	0.046	✔	✔	✗	✗	✗
Bars and restaurants close	0.186	✔	$\bigtriangleup$	✗	✗	✗
Schools close	0.186	✔	$\bigtriangleup$	✗	✗	✗
Tertiary education facilities close	0.093	✔	$\bigtriangleup$	✗	✗	✗
Small food retailers close	0.093	✔	✗	✗	✗	✗
Non-essential retail business close	0.093	✔	$\bigtriangleup$	✗	✗	✗
Summation		2.184	1.673	1.116	0.930	0
Base reproduction number ($R_0$)		2.5	2.5	2.5	2.5	2.5
Final R value		0.316	0.827	1.384	1.570	2.5

We identified which interventions are applied in the New Zealand alert levels, indicated in Table 2 by a tick (✔) or a cross (✗) to capture if a given intervention is applied (respectively not applied) in a given alert level. Each intervention is also weighted in its effectiveness, with the weighted sum being 2.184. A triangle ($\bigtriangleup$) is used when an intervention is partially applied. In this case, half the weight is considered. At the bottom of Table 2, we show the calculated reproduction numbers for each alert level by taking into account both the base reproduction number $R_0$ and the interventions applied. In summary, the $R_0$ values for alert levels 4 through 1 are 0.316, 0.827, 1.384, 1.570 respectively. The maximum value of $R_0$ is 2.5, which corresponds to level 0. Additionally, for use with the previously described PL-2 model, we impose an $R_0$ value of 0.02 for confirmed cases to capture the enforced isolation placed upon them.

In order to capture the increased testing rates (and more rigorous contact tracing) associated with higher alert levels, we also vary the value *c* used to represent the testing rate in PL-3. In this model, we use daily rates of 0.3, 0.2, 0.15 and 0.01 for alert levels 4 through 1, respectively.

The controller NZ-C2, in Fig. 2a, matches a given alert level to its corresponding $R_0$ value. Initially, the control starts from Pre-LD and move to LD just like the previous controller in Fig. 1c. After 33 days this corresponds to 27th April 2020, which is the scheduled start of Level 3. After this point, the control enters Level 3, and the reproduction number is set to 0.827. The transitions $\textcircled {1}$ through $\textcircled {6}$ are taken based on time, like NZ-C1, since these mimic known time based government actions.

Subsequently, the government decisions, which are yet unknown, will have to be mimicked using more complex mechanisms. We use the following strategy to determine the transition conditions, which will not be time based alone, as follows. First we denote the rate of new cases per day as ${\dot{C}}$ and the current number of infected cases is $I_c$ We consider the following parameter values based on the published data from New Zealand^14^. We set $k_{l4}$, $k_{l3}$, $k_{l2}$, and $k_{l1}$ as 100, 50, 5, and 1, respectively. $dk_{l3}$, $dk_{l2}$, and $dk_{l1}$ are 10, 5, and 0.01, respectively. Finally, in our results, we assume that a vaccine will arrive 365 days after 20th March 2020. At this time, the number of susceptible people progressively decreases to zero, assuming widespread adoption of an effective vaccine.

The conditions for increasing the alert level are based on the current number of infected cases ($I_c$). For example, from level two if $I_c \ge k_{l3}$ then the alert level immediately rises to three. On the other hand, the alert level can go down if the increasing rate of new cases per day (${\dot{C}}$) is less than a certain amount. For example, from level three if ${\dot{C}} \le dk_{l3}$ then the alert level decreases to level two. In addition, to avoid frequent oscillations between levels, a minimum duration within a level before being able to drop down to a lower level is added, and is set to be 14 days.

### The Italy model of COVID-19

Unlike New Zealand, Italy does not issue a systemic intervention strategy for COVID-19. Instead, the government releases the actions incrementally as they are needed. The Oxford COVID-19^15^ provides a stringency index of the measures taken by various governments around the world. According to the stringency index of Italy’s interventions, we divide the transmission trajectory into four phases. Considering that the initially reported cases are mostly imported rather than community transmission, the starting point of the analysis is 23rd February 2020, when the reported number of cases is 155. As a first attempt, we use the SEIR model^14^ and curve fitting to estimate policy-specific reproduction numbers for Italy. We use the MATLAB function lsqcurvefit to search for these reproduction numbers for each phase by minimizing the square of the residual error between the SEIR simulation and the reported data^48^. The resulting reproduction numbers are listed in Table 3.

**Table 3:** Estimated reproduction numbers for each phase in Italy.

Phase	Phase 0	Phase 1	Phase 2	Phase 3	Phase 2*
Date range	23 Feb–4 Mar	4 Mar–10 Mar	10 Mar–20 Mar	20 Mar–27 Jul	27 Jul–28 Aug
$R_0$	6.3533	4.8051	3.2704	0.5808	2.6472

The controller is shown in Fig. 3a. For dropping alert levels, we have values of 10, 5, and 0.01 for $dk_{l3}$, $dk_{l2}$, and $dk_{l1}$ respectively. The population of Italy (*N*) is 60,461,828 and we have level changing constraints of 6046,3023, and 605 for $k_{l3}$, $k_{l2}$, and $k_{l1}$, respectively.

### HAML

Hybrid Automata Modelling Language (HAML)^23^ is a recently developed tool in our group for the compositional modelling and verification of CPSs. To create the CCPE system in HAML we simply create two automata, one each for the plant and controller, and compose them as a single network. For the plant model, Listing 1a, we have an automata with an input $R_0$ value which is used to determine the rate of reproduction in the model. Additionally, there are two outputs for the number of currently infected (and tested) people, $I_c$, and the rate of change in the number of cases ($C_{dot}$). The two locations of Fig. 1b are shown which have the same flow constraints but differ in their calculation of the CFR to create a piecewise implementation of Eq. (10) through the use of update constraints.

The discrete controller has external inputs and outputs which mirror those of the plant model, having two inputs, $I_c$ and $C_{dot}$, and a single output, $R_0$. Listing 1b shows this controller captured in HAML, using locations for each of discrete modes that it can be in. When the number of current confirmed cases ($I_c$) reaches an upper bound for each location then the control progresses to a higher alert level, while when the change in number of cases ($C_{dot}$) reaches a lower bound then control transitions to a lower alert level. The values of $R_0$ for each control location are taken from Table 2.

Finally, composition between these two components simply requires mapping their respective inputs and outputs together. This is achieved by defining each of the previous models, creating a single instance for each, and then providing the mapping of their variables, as shown in Listing 1c.

**Listing 1:**
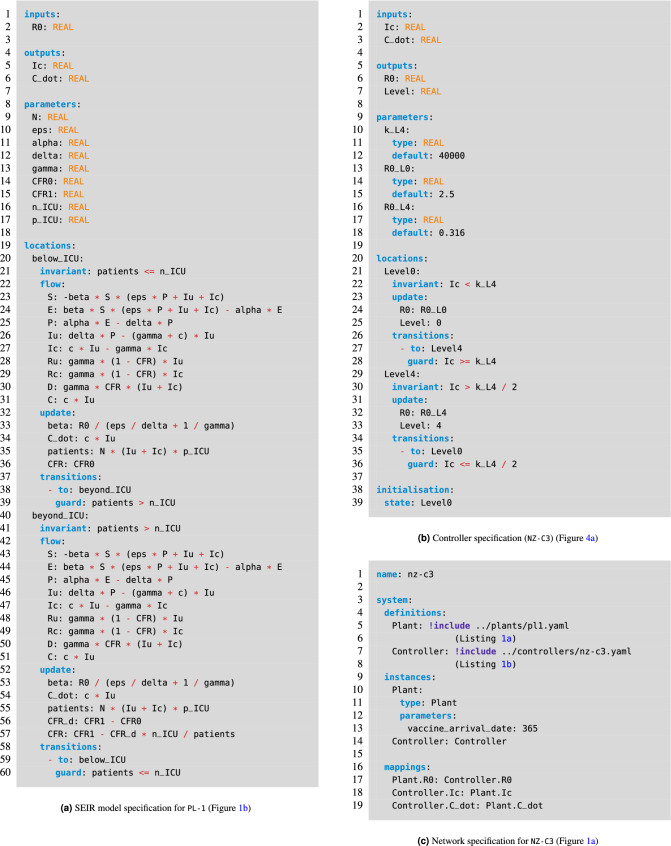
Example HAML specifications for the Compositional Cyber-Physical Epidemiology system.

## Data Availability

*Accession codes* All models and code used for this work are publicly available on GitHub: https://github.com/PRETgroup/ccpe-covid19. The real-world data used to validate our models was obtained from the publicly available dataset of Johns Hopkins CCSE: https://github.com/CSSEGISandData/COVID-19.
